# Metabolic and Cognitive Effects of Ranolazine in Type 2 Diabetes Mellitus: Data from an in vivo Model

**DOI:** 10.3390/nu12020382

**Published:** 2020-01-31

**Authors:** Velia Cassano, Antonio Leo, Martina Tallarico, Valentina Nesci, Antonio Cimellaro, Teresa Vanessa Fiorentino, Rita Citraro, Marta Letizia Hribal, Giovambattista De Sarro, Francesco Perticone, Giorgio Sesti, Emilio Russo, Angela Sciacqua

**Affiliations:** 1 Department of Medical and Surgical Sciences, Magna Græcia University, 88100 Catanzaro, Italy; 2 Science of Health Department, Magna Græcia University, 88100 Catanzaro, Italyvnesci@unicz.it (V.N.);; 3 Pugliese-Ciaccio, Hospital, Internal Medicine Unit, 88100 Catanzaro, Italy; 4 Department of Clinical and Molecular Medicine, University of Rome-Sapienza, 00189 Rome, Italy

**Keywords:** Type 2 Diabetes, Type 3 diabetes, Ranolazine, Metformin, Cognitive impairment, Alzheimer’s disease

## Abstract

Type 2 diabetes mellitus (T2DM) is a risk factor for cognitive impairment. Ranolazine, an anti-ischemic drug used in the treatment of angina pectoris, has been shown to possess hypoglycemic properties in pre-clinical and clinical studies. The aim of this study was to evaluate the effects of ranolazine on glucose metabolism and cognitive function in a T2DM model of Wistar rats. Diabetes was induced by a high fat diet (HFD) and streptozotocin (STZ). The control group received a normal caloric diet (NCD) and sodium citrate buffer. Metformin, an effective hypoglycemic drug, was employed as a positive control. Animals were divided into the following groups: HFD/STZ + Ranolazine, HFD/STZ + Metformin, HFD/STZ + Vehicle, NCD + Vehicle, NCD + Ranolazine, and NCD + Metformin. Rats received ranolazine (20 mg/kg), metformin (300 mg/kg), or water, for 8 weeks. At the end of the treatments, all animals underwent to an intraperitoneal glucose tolerance test (IPGTT) and behavioral tests, including passive avoidance, novel object recognition, forced swimming, and elevate plus maze tests. Interleukin-6 plasma levels in the six treatment groups were assessed by Elisa assay. Body mass composition was estimated by nuclear magnetic resonance (NMR). Glucose responsiveness significantly improved in the HFD/STZ + Ranolazine (*p* < 0.0001) and HFD/STZ + Metformin (*p* = 0.003) groups. There was a moderate effect on blood glucose levels in the NCD + Ranolazine and NCD + Metformin groups. Lean body mass was significantly increased in the HFD/STZ + Ranolazine and HFD/STZ + Metformin animals, compared to HFD/STZ + Vehicle animals. Ranolazine improved learning and long-term memory in HFD/STZ + Ranolazine compared to HFD/STZ + Vehicle (*p* < 0.001) and ameliorated the pro-inflammatory profile of diabetic mice. These results support the hypothesis of a protective effect of ranolazine against cognitive decline caused by T2DM.

## 1. Introduction

Type 2 diabetes mellitus (T2DM) is a public health problem that affects millions of people all over the world. T2DM is characterized by insulin resistance associated with a progressive decline of pancreatic β-cell function [1]. T2DM is also linked to an increased risk of cardiovascular disease and cardiovascular comorbidities [2]. Recent data show that patients with T2DM have a higher risk of Alzheimer’s disease (AD) than patients without T2DM; altered insulin secretion and insulin resistance seems to be the link between T2DM and AD [3,4]. It has also been reported that T2DM may cause brain insulin resistance, oxidative stress, and cognitive impairment. Extensive disturbances in brain insulin and insulin like growth factor 1 (IGF-1) signaling mechanisms represent early and progressive abnormalities and could account for the majority of the molecular, biochemical, and histopathological lesions in AD [5]. The common background of the two diseases has led to the term type 3 diabetes being generated, referring to AD as a form of diabetes that selectively involves the brain [5]. In patients with diabetes mellitus, the hypothalamic–pituitary–adrenal axis is hyperactive, and this can cause high level of steroid hormones. A previous study has demonstrated that in both insulin-deficiency and insulin-resistance, diabetes damages hippocampus-dependent memory, and high levels of adrenal steroid corticosterone contribute to these adverse effects; rats treated with streptozotocin have reduced levels of insulin and disablement in hippocampal-neurogenesis, synaptic plasticity, and learning [6]. Despite this strong evidence, established guidelines for the treatment of individuals with both diseases are unfortunately still lacking [7]. Recently, the cognitive dysfunction sub-study of the large CARMELINA international trial has shown that the dipeptidyl peptidase 4 inhibitor linagliptin was unable to prevent cognitive decline in 1545 subjects with T2DM and cardiorenal disease, notwithstanding its effectiveness in improving glycemic control [8]. These results underline the need to characterize the molecular mechanisms linking cognitive dysfunction and T2DM and to explore possible novel pharmacological approaches.

Ranolazine is an active piperazine, approved by Food and Drug Administration (FDA) in 2006 for the treatment of chronic angina pectoris and as an anti-arrhythmic. It has been demonstrated by electrophysiology studies that ranolazine displays anti-arrhythmic properties by blocking late sodium currents (INaL) over peak fast sodium currents (INa). Ranolazine decreases calcium influx through the sodium/calcium exchanger by blocking INaL, making it an effective treatment for chronic angina [9]. Ranolazine also reduces repolarization in long-QT mutations, decreases the transmural dispersion of repolarization across the ventricular wall, and reduces the risk factors for ventricular tachycardia and arrhythmia [10]. Peak plasma levels of ranolazine occur 4–6 h after an oral dose, with 50%–55% bioavailability. In addition to its anti-ischemic and antianginal effects, ranolazine has been shown to lower hemoglobin A1c in patients with coronary artery disease and type 2 diabetes mellitus in two clinical studies [11,12].

In the CARISA trial (Combination Assessment of Ranolazine in Stable Angina), ranolazine treatment for 12 weeks reduced HbA1c levels by 0.7 ± 0.18% in patients with chronic angina (189 with T2DM and 634 subjects without the disease) regardless of concomitant insulin or oral hypoglycemic therapy [11]. In the Merlin TIMI 36 trial (Metabolic Efficiency with Ranolazine for less Ischemia in Non-ST-Elevation Acute Coronary Syndrome Thrombolysis in Myocardial Infarction 36) ranolazine, added to standard therapy, reduced HbA1c by 0.64% in 2829 individuals with T2DM, and by 0.12 in 1477 non-diabetic subjects [12]. Ranolazine may exert its hypoglycemic effects throughout the inhibition of glucagon secretion from pancreatic islets, as it blocks Na channels (Nav 1.3 isoform) in α-cells [13]. In non-clinical studies, ranolazine was found to lower fasting and non-fasting glucose levels and to preserve pancreatic β-cells in streptozotocin-induced diabetic mice [14].

It has also been observed that mice treated with ranolazine had healthier islet morphology and higher β-cell mass compared with the vehicle-treated group. The number of apoptotic islet cells was also significantly reduced in the pancreata of ranolazine-treated mice. Long-term treatment with ranolazine resulted in β-cell preservation and enhanced insulin secretion [14].

Under the hypothesis that ranolazine could act as neuroprotective drug, several studies have evaluated its effects on the central nervous system (CNS). In a study carried out on primary cultures of astrocytes and neurons, incubated for 24 h with ranolazine, the treatment increased cell viability and proliferation. Ranolazine also increased anti-inflammatory PPAR-γ protein expression and reduced pro-inflammatory proteins, interleukin- 1 β, and Tumor Necrosis Factor- α, levels [15]. Therefore, it is possible to hypothesize that ranolazine could act as a neuroprotective drug in the CNS by promoting astrocyte viability and preventing necrosis and apoptosis.

The aim of the present study is to evaluate the effects of ranolazine on glucose metabolism and cognitive function in a T2DM model of Wistar rats.

## 2. Materials and Methods

### 2.1. Animals

All the experiments were carried out on male Wistar rats (*n* = 48; *n* = 8 per group) weighing about 200–240 g. Wistar rats progenitors were initially purchased from Charles River Laboratories (Calco, Lecco, Italy) and the rats used in these protocols were all obtained from our breeding colony at the University of Catanzaro animal facility, as previously described [16,17]. Wistar rats were housed 2/3 per cage and kept under stable environmental conditions, humidity (60 ± 5%), and temperature (21 ± 2 °C), in a room with a 12/12-h reversed light/dark cycle (lights on at 20:00). Rats received food and drinking water ad libitum. Procedures involving animals and their care were performed in agreement with the international and national law and policies (EU Directive 2010/63/EU for animal experiments, ARRIVE guidelines, and the Basel declaration, including the 3 R concept). The experimental protocols and the methods described herein were approved by the Animal Care Committee of the University of Catanzaro, Italy. All efforts were made to minimize animal suffering and to decrease the number of animals used.

### 2.2. Drugs

Streptozotocin (STZ), obtained from Sigma Aldrich Milan Italy, was dissolved in 0.01 M sodium citrate buffer (pH 4.5) and administrated i.p. at a dose of 35 mg/kg body weight (bw) [18]. Ranolazine, obtained from Menarini Industrie Farmaceutiche Riunite, was dissolved in 10% solution of dimethylsulphoxide (DMSO) and administered at 20 mg/Kg per o.s. [19], whereas metformin, obtained from Teva Pharmaceutical Industries, was dissolved in water and administered at 300 mg/Kg per o.s. [20].

### 2.3. Induction of Diabetes and Pharmacological Treatment

Wistar rats (*n* = 48) were randomly divided into two groups: Type 2 diabetes mellitus model group and control (vehicle) group. Diabetes was induced by feeding the animals (*n* = 24) with a high fat diet (HFD: 59% fat, 15% protein, 20% carbohydrates), obtained from Laboratorio Piccioni S.R.L. Gessate Italy, for 50 days, and by administering two low doses of STZ (35mg/kg i.p), at day 21 and day 42 from the beginning of dietary manipulation, according to a previously established protocol [21]. Likewise, the control group (*n* = 24) was fed with a normal caloric diet (NCD) and injected at day 21 and day 42 from the beginning of dietary manipulation with 0.01 M sodium citrate buffer (pH 4.5).

Diabetes induction was verified eight days after the second dose of STZ, by the intraperitoneal glucose (2 mg/kg) tolerance test (IPGTT). Briefly, rats were fasted for 6 h before IPGTT and blood samples were collected from tail vein in fasted animals at the following time points: T0 (before glucose injection) and 30, 60, 90, and 120 min after glucose injection. Blood glucose levels were measured with an Accu-Chek Aviva glucometer (Coefficient of variation (CV), at 24% for values at or above 100 mg/dL; Hoffman-La Roche, Basel, Switzerland). The treated rats were scored as diabetic once the blood glucose levels reached > 250 mg/dL [22].

Subsequently, diabetic rats were randomly divided into 3 groups (*n* = 8 for group): HFD/STZ + Vehicle (DMSO), HFD/STZ + Ranolazine, HFD/STZ + Metformin. Rats from the NCD control group were randomly assigned to NCD + Vehicle (DMSO) group, NCD + Ranolazine, and NCD + Metformin groups (*n* = 8 for group). In detail, rats received either ranolazine (20 mg/kg) or metformin (300 mg/kg) orally for 8 consecutive weeks. Drugs were administered in the drinking water by dissolving the desired dose into 120 mL tap water, as previously described [23]. The drug dose was calculated based on previous evidence that rats drink 12 mL/100g/day; this was subsequently verified by checking the volume actually drunk in each cage. Water bottles were wrapped in silver foil to protect from light and solutions were freshly prepared and replaced 3 times a week [23,24]. 

Body composition (fat mass, lean mass, free, and total water) was recorded using the nuclear magnetic resonance spectroscopy device, EchoMRI-700 (Echo Medical Systems, Houston, TX, USA), before and after induction of diabetes, as well as at the end of pharmacological treatments.

Metabolic evaluations at the end of the 8-weeks of treatment were carried out by investigators blinded to group allocation.

### 2.4. Interleukin-6 Detection

Rats were anesthetized using tiletamine/zolazepam 50 mg/kg i.p. (1:1; Zoletil 100; VIRBAC S.r.l., Milan Italy) to collect blood from their ophthalmic plexuses. Blood was collected in tubes without anticoagulant for interleukin 6 (IL-6) quantification from serum. Blood was centrifuged at 4000 rpm for 15 min and serum was separated and stored at −20°C. Serum levels of IL-6 were determined by an enzyme-linked immunosorbent assay (ThermoFisher Scientific, Monza, Italy), according to the manufacturer’s protocol. The inter and intra assay CV were respectively 10% and 5%; all samples were assessed in duplicate. Results are expressed as fold variation over control. 

### 2.5. Behavioral Tests

In order to reduce the number of rats used, all experimental groups were subjected to several tests. When the same animal was subjected to several tests, at least one day (range one to three days) was allowed for recovering, as previously reported [25]. All behavioral tests were carried out under controlled environmental conditions, such as temperature, humidity, and light intensity (dim illumination), and all tests, except for passive avoidance, were performed with the support of video-tracking software (EthoVision XT8; Noldus Technology, Wageningen, the Netherlands). In order to avoid possible circadian modifications of the test results, all experiments were carried out between 9.00 am and 11.00 am [16]. In order to eliminate olfactory cues, both mazes and apparatuses were systematically cleaned.

### 2.6. Passive Avoidance (PA)

Passive avoidance (PA) is a fear-motivated test used to study learning and memory in rodents. In this test, rodents learn to restrain their innate tendency, namely preferring a dark compartment rather than an illuminated one [26]. Passive avoidance behavior was assessed by a step-through type apparatus (Ugo Basile, Italy, model 40550), measuring 57 × 27 × 30 cm, which consisted of a cage divided into two compartments (light and dark) by a sliding door. The PA test was conducted over two consecutive days, as previously reported [16]. Briefly, on day one (habituation), rats were placed individually in the illuminated compartment and allowed to freely scour for 5 min. The conditioning trial was started 15 min following habituation. During this trial, rats were individually entered into the illuminated compartment. Subsequently, after a delay of 30 sec, the sliding door was automatically opened; when rats passed into the dark compartment, the sliding door was automatically closed, and an electrical foot shock was delivered via the floor grid (0.5 mA for 3 s). Each rat had 300 sec to enter in the dark compartment. The latency to enter (s) in the dark compartment was recorded and analyzed. The retention trial was carried out 24 h after the conditioning trial by re-introducing the rat into the light compartment of the cage and by recording time taken to enter into the dark compartment. No foot shock was delivered in this trial. Retention memory is directly linked to the latency to enter into the dark compartment: The better the memory, the greater the latency [25,27]. 

### 2.7. Novel Object Recognition Test (nORT)

The novel object recognition test is based on the tendency of rodents to discover novel objects [28]. The nORT was carried out, as previously reported by Bartolini and colleagues, with some minor modifications [29]. Rats, individually located in an open field Plexiglas box (70 × 60 × 30 cm), were trained to distinguish between different shaped objects (cubes, pyramids, and cylinders) [30]. Briefly, on day one, rats were subjected to the habituation trial, in which they could freely explore the arena for 6 min. On day two, a single session of two trials (T1 and T2), separated by a retention interval of 60 min, was performed. In detail, during the familiarization trial (T1) two identical objects were placed in two opposite corners of the box. Each rat was placed in the center of the box, facing away from the objects and left to freely investigate objects for a maximum of 5 min. T1 ended when rat achieved 20 s of exploration for each object. Exploration was defined as directing the nose at a distance < 2 cm to the object and/or touching object with the nose. During T2, a novel object with different shape and color was substituted for one of the familiar objects presented in Tl and rats were left in the box for 5 min. After this session, the rats returned to their home cage. The time spent for the exploration of the familiar (F) and the novel object (N) was recorded separately and the difference between the two exploration times was taken as the discrimination index (DI = N − F/N + F). Moreover, to prevent place preference, the role (F or N) and the location of the objects during T2 were randomly changed. Total distance moved (cm) were also assessed and analyzed for every experimental group in order to detect locomotor impairment [16].

### 2.8. Forced Swimming Test (FST)

The forced swimming test, despite some limitations, is widely used to test depressive-like behavior and to screen antidepressant drug efficacy in rodents [31]. In detail, we performed a FST protocol previously standardized in our laboratory [24,32]. Briefly, rats were individually placed for 6 min into a Plexiglas cylinder (height 47 cm, diameter 38 cm) filled with water and maintained at 23–25 °C. The immobility time (IT), related with depressive-like behavior, was measured during the last 4 min of the 6 min testing period. Afterwards, rats were dried and then normally housed. The condition for immobility time/passive swimming (IT) was floating vertically in the water, while making only the movements required to keep the head above the surface of the water. Mean swimming velocity and total distance moved were also evaluated for every experimental group in order to avoid the effects of potential locomotor impairment [24,32].

### 2.9. Elevate Plus Maze (EPM)

Elevated plus maze, used to assess anxiety-like behavior in rodents, consists of two opposing open arms (50 × 10 cm) and two opposing closed arms of the same size with 40 cm high walls. The arms were linked to a central square (10 × 10 cm) and elevated 80 cm above the floor. Rats were positioned in the central square facing a closed arm. The number of entries into, and the time spent on each arm and central square were recorded for 10 min. Less time spent in open arms and in central square revealed anxious-like behavior, and vice versa. Mean velocity and total distance moved were also assessed for every experimental group [25,27].

### 2.10. Statistical Analysis

For the analysis of metabolic parameters, data are expressed as mean ± SD or as mean ± SEM and comparisons were performed using SPSS 20.0 statistical software (SPPS, Inc., Chicago, IL, USA). For all variables, comparison between baseline and 8 weeks of treatment were performed using paired Student’s *t* test. The area under the curve (AUC) under the IPGTT curve was calculated with the trapezoidal formula as follows 0.25× (glycemia T0+ glycemia T120) +0.5× (glycemia T30+ glycemia T60+ glycemia T90). We also considered the difference in Δ values between baseline and 8 weeks of treatment for the following parameters: Blood glucose levels during IPGTT and body mass composition. The ANOVA test was used to test the differences between groups, and the Bonferroni’s post hoc analysis was performed for multiple comparisons. A value of *p* ≤ 0.05 was considered significant.

For behavioral parameters and to compare IL-6 serum levels, statistical analyses were carried out using GraphPad Prism 8.0 (GraphPad Software, Inc., La Jolla, CA 92037, USA). We performed two-way ANOVA followed by Tukey’ s post hoc test to analyze and compare behavioral data, with experimental model (two levels) and treatment (vehicle or drugs) as factors. Results are expressed as mean ± SEM. All tests used were two sided and a value of *p* ≤ 0.05 was considered significant.

## 3. Results

### 3.1. Blood Glucose Levels and Body Composition

**Figure 1:**
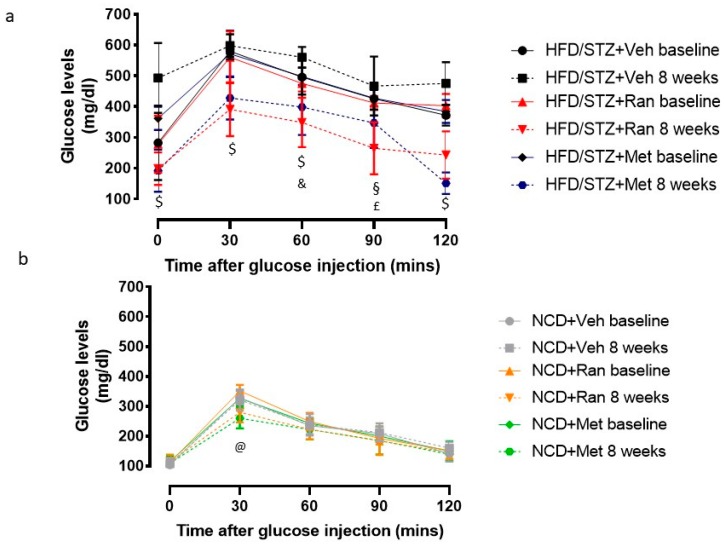
Blood glucose levels (mg/dl) during the intraperitoneal glucose tolerance test (IPGTT) at baseline (after induction of diabetes) and after 8 weeks of treatment in diabetic (**a**) and normal caloric diet (NCD) (**b**) rats. Values are means ± SD. $ *p* < 0.0001 HFD/STZ + Ranolazine and HFD/STZ + Metformin vs. HFD/STZ + Vehicle, § *p* = 0.0005 HFD/STZ + Ranolazine vs. HFD/STZ + Vehicle; & *p* = 0.0003 HFD/STZ + Metformin vs. HFD/STZ + Vehicle; £ *p* = 0.017 HFD/STZ + Metformin vs. HFD/STZ + Vehicle; @ *p* = 0.03 NCD + Ranolazine and NCD + Metformin vs. NCD + Vehicle. HFD = High fat diet; NCD = Normocaloric diet; STZ = Streptozotocin; Veh = Vehicle; Ran = Ranolazine; Met = Metformin.

To evaluate ranolazine effect on glucose homeostasis, all animals were subjected to IPGTTs at the end of the 8 weeks of treatment. As shown in Figure 1a, fasting blood glucose levels and glucose tolerance significantly worsened in the HFD/STZ + Vehicle group as the rats aged and continued to assume an HFD (black lines Figure 1a, *p* < 0.05 T0 and T30, *p* < 0.0001 T120). By contrast, glucose response during IPGTT significantly improved in HFD/STZ + Ranolazine group (red lines, Figure 1a, *p* < 0.05 T0, T30, T6, *p* < 0.0001 T90 and T120) and in HFD/STZ + Metformin group (blue lines, Figure 1a, *p* < 0.05 T0, T30, T60). Glucose response curves of HFD/STZ + Ranolazine and HFD/STZ + Metformin animals at 8 weeks were significantly better than the curves of HFD/STZ + Vehicle rats (*p* < 0.0001 by two-way ANOVA, *p* < 0.0001 by Student’s *t* test in all single comparisons, except T60 HFD/STZ + Metformin vs. HFD/STZ + Vehicle *p* = 0.0003, T90 HFD/STZ + Metformin vs. HFD/STZ + Vehicle *p* = 0.017, and T90 HFD/STZ + Ranolazine vs. HFD/STZ + Vehicle *p* = 0.0005). No changes were otherwise detected in the NCD group (Figure 1b, grey lines), while ranolazine and metformin had a moderate effect on blood glucose levels (Figure 1b, orange and green lines), with the only statistically significant differences at T30 (*p* = 0.03 for both NCD + Ranolazine and NCD + Metformin vs. NCD + Vehicle).

**Figure 2:**
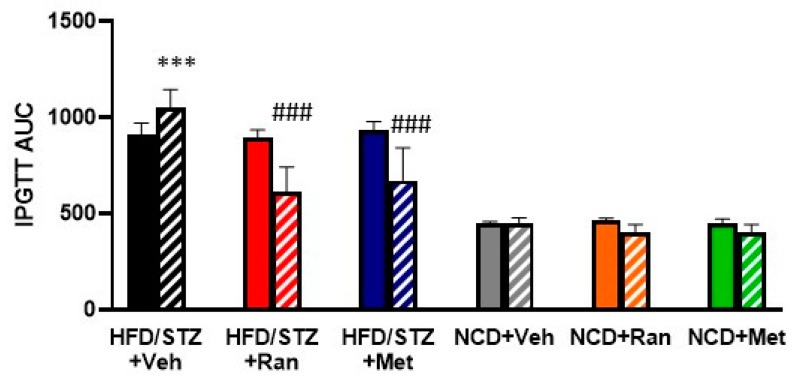
Area under the curve (AUC) during a 120 min IPGTT at baseline (full bars) and after 8 weeks (striped bars) of treatment. Values are expressed as mean ± SD; *** *p* < 0.0001 significantly different from NCD + Vehicle group, ### *p* < 0.0001 significantly different from HFD/STZ + Vehicle group by two-way ANOVA with Tukey’ s post hoc test. AUC = Area under curve; HFD = High fat diet; NCD = Normocaloric diet; STZ = Streptozotocin; Veh = Vehicle; Ran = Ranolazine; Met = Metformin. 0.011 NCD 0.0002 met.

To confirm the significance of the observed changes, we calculated the AUC during the 120 min of IPGTT for the six experimental groups. The IPGTT-AUC was significantly increased in the HFD/STZ + Vehicle group after 8 weeks of treatment compared to baseline. Moreover, the IPGTT-AUC was significantly decreased in the HFD/STZ + Ranolazine group, HFD/STZ + Metformin group, NCD + Ranolazine group, NCD + Metformin group, compared to their respective baseline. No changes were detected for the NCD + Vehicle group (Figure 2). The IPGTT-AUC of the ranolazine and metformin treated groups were significantly different from those of the respective vehicle-treated control groups.

In addition, changes in blood glucose levels, expressed as Δ variation between baseline and 8 weeks of treatment, were significantly different (*p* < 0.0001 by ANOVA) for all time points of IPGTT in the three groups with diabetes. In the Bonferroni’s post hoc analysis, HFD/STZ + Vehicle vs. HFD/STZ + Ranolazine and HFD/STZ + Vehicle vs. HFD/STZ + Metformin, the *p* value was <0.0001 for all time points, except for ΔT30 (*p* = 0.001) and ΔT60 (*p* = 0.037) HFD/STZ + Vehicle vs. HFD/STZ + Metformin. Instead, in the comparison between HFD/STZ + Ranolazine and HFD/STZ + Metformin, the *p* value was >0.05 for all time points of IPGTT. By contrast, in the ANOVA test performed between the three control groups, *p* values were always >0.05.

**Figure 3:**
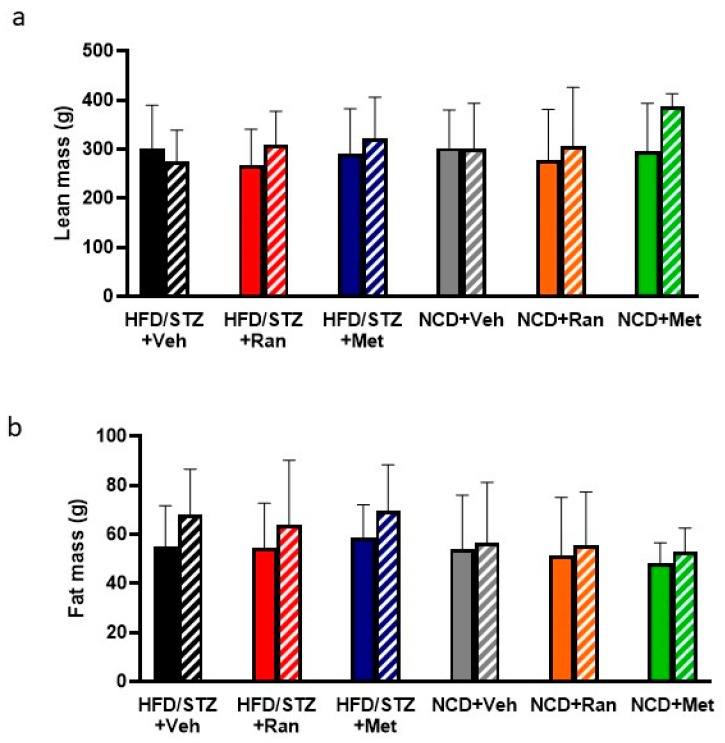
Comparison of lean (**a**) and fat (**b**) mass, at baseline (full bars) and after 8 weeks (striped bars) of treatment in each experimental group. Values are mean ± SD; HFD = High fat diet; NCD = Normocaloric diet; STZ = Streptozotocin; Veh = Vehicle; Ran = Ranolazine; Met = Metformin.

During the 8-weeks, we did not detect any change in food or drink intake. By nuclear magnetic resonance (NMR), a significant increase was observed in the lean mass of HFD/STZ + Ranolazine group and of HFD/STZ + Metformin group (Figure 3a). No significant changes were detected in the body composition of the NCD + Vehicle group (Figure 3a,b). A significant increase in the lean mass was detected also in NCD + Ranolazine (*p* = 0.007) and NCD + Metformin rats (*p* = 0.02) (Figure 3a), as compared to their respective baseline. On the contrary, in the HFD/STZ + Vehicle group, there was a significantly increase in fat mass, while there were no changes in lean mass (Figure 3b). When comparing the effects across treatments, there were, however, no statistically significant differences. 

### 3.2. IL-6 Serum Levels

**Figure 4:**
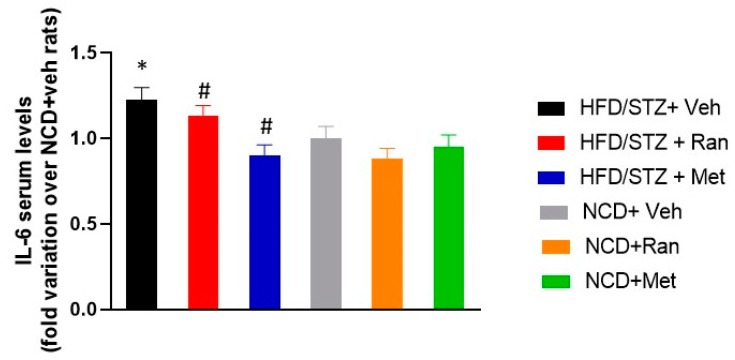
IL-6 serum levels in each experimental group. Data values are mean ± SD and are expressed as fold variation over NCD + Vehicle rats. * *p* < 0.05 significantly different from NCD + Vehicle group. # p<0.05 significantly different from HFD/STZ + Vehicle group by two-way ANOVA with Tukey’ s post hoc test. HFD = High fat diet; NCD = Normocaloric diet; STZ = Streptozotocin; Veh = Vehicle; Ran = Ranolazine; Met = Metformin.

To explore the possibility that ranolazine may improve the inflammatory profile of diabetic mice, we assessed serum levels of the key pro-inflammatory cytokine IL-6. As shown in Figure 4, IL-6 serum concentration in the HFD/STZ + Vehicle group was markedly higher as compared to the NCD + Vehicle group (*p* < 0.05). Notably, the treatment of diabetic rats with ranolazine or metformin significantly decreased the elevated serum levels of IL-6 (*p* < 0.05).

### 3.3. Passive Avoidance (PA)

**Figure 5:**
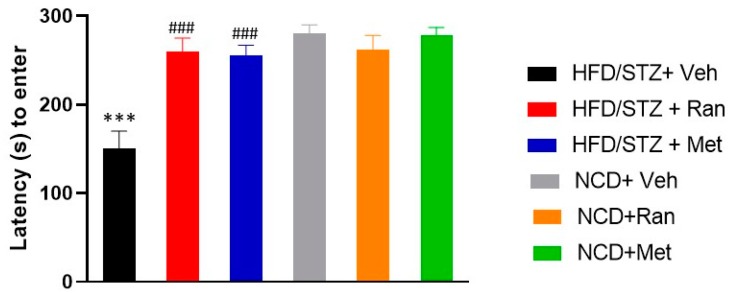
Passive avoidance test (PA). Bars indicate the latency (s) time to enter in the dark chamber during the retention session. Data values are means ± SEM. *** *p* < 0.0001Significantly different from NCD/STZ vehicle group. ### *p* < 0.0001significantly different from HFD/STZ vehicle group by two-way ANOVA with Tukey’ s post hoc test. NCD = Normocaloric diet; STZ = Streptozotocin; Veh = Vehicle; HFD = High fat diet; Ran = Ranolazine; Met = Metformin.

Learning and memory performance were evaluated in the passive avoidance test. There was not a significant (*p* > 0.05) difference in the latency (s) to enter in the dark compartment, during the conditioning trial, among groups (data not shown). Conversely, data revealed a significant interaction between the T2DM experimental model and drugs treatment on the latency to enter (s) in the dark compartment during the retention session (F_(2,42)_ = 9.14; *p* = 0.0005; *n* = 8 per group; Figure 5). In detail, HFD/STZ rats had a significantly shorter latency (s) to enter in the dark compartment, during the retention session, in comparison to NCD/vehicle rats, indicating poorer learning and memory performance (*p* < 0.0001). However, treatment either with ranolazine or metformin to HFD/STZ rats induced a significant improvement of their performances (*p* < 0.0001), with latency to enter in the dark compartment being reported nearly at NCD-fed rats’ level. At odds, there was no significant difference between the magnitude of ranolazine and metformin effect on latency (s) to enter in the dark compartment in HFD/STZ rats. Likewise, treatment with either ranolazine or metformin did not significantly (*p* > 0.05) modify this parameter in NCD-fed rats (Figure 5). 

### 3.4. Novel Object Recognition Test (nORT)

**Figure 6:**
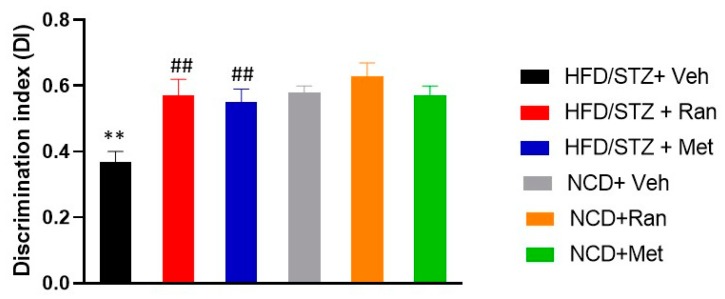
novel Object recognition test (nORT). Bars indicate the Discrimination Index (s). Data values are means ± SEM. ** *p* < 0.01 significantly different from NCD/STZ vehicle group. ^##^ *p* < 0.01 significantly different from HFD/STZ vehicle group by two-way ANOVA with Tukey’s post hoc test. NCD = Normocaloric diet; STZ = Streptozotocin; Veh = Vehicle; HFD = High fat diet; Ran = Ranolazine; Met = Metformin.

The nORT was performed to study working-memory in each experimental group. No significant (*p* > 0.05) difference was found among experimental groups in the ability to reach 20s of exploration in the familiarization trial. At odds, data showed a significant interaction between experimental model and drugs treatment on Discrimination Index (DI) (F_(2,42)_ = 4.64; *p* = 0.015; *n* = 8 per group; Figure 6). In details, HFD/STZ rats had a significant (*p* = 0.004) reduction of DI in comparison to NCD-fed group, suggesting a working memory impairment. Interestingly, treatment either with ranolazine or metformin was able to induce a significant (*p* = 0.001 and *p* = 0.003, respectively) increase of DI in HFD/STZ rats, rescuing the observed memory impairment. Additionally, there was no significant (*p* > 0.05) difference between the magnitude of effect of the two treatments on DI in HFD/STZ rats. Likewise, treatment either with ranolazine or metformin did not significantly (*p* < 0.05) change memory performance in NCD-fed rats (Figure 6). Mean velocity and total distance moved did not differ among groups (data not shown).

### 3.5. Forced Swimming Test (FST) and Elevated Plus Maze (EPM)

**Figure 7:**
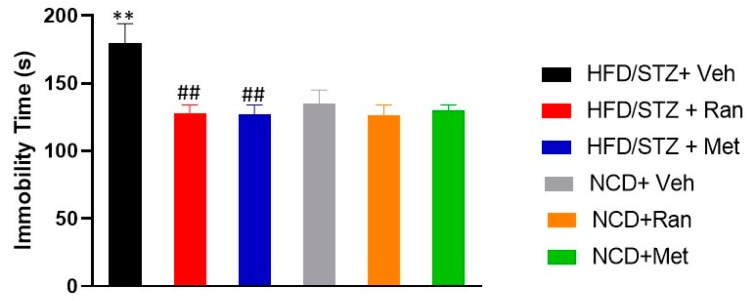
Forced swimming test (FST). Bars indicate the immobility time (s). Data values are means ± SEM. ** *p* < 0.01 significantly different from NCD/STZ vehicle group. ^##^ *p* < 0.01 significantly different from HFD/STZ vehicle group by two-way ANOVA with Tukey’ s post hoc test. NCD = Normocaloric diet; STZ = Streptozotocin; Veh = Vehicle; HFD = High fat diet; Ran = Ranolazine; Met = Metformin.

Depressive-like behavior was evaluated in the FST. Our data showed a significant interaction between experimental model and drugs treatment on immobility time (s) (F_(2,42)_=4.4; *p* = 0.017; *n* = 8 per group; Figure 7). In details, HFD/STZ rats had a significantly (*p* < 0.009) increased immobility time (IT) (sign of depressive-like behavior) in comparison to the NCD-fed group. Both ranolazine and metformin significantly reduced (*p* = 0.0037 and *p* = 0.0039, respectively) the IT in HFD/STZ rats, reporting it to levels comparable to those of the NCD-fed group. Likewise, treatment either with ranolazine or metformin did not significantly (*p* < 0.05) change IT in NCD-fed rats. The magnitude of treatments effect on IT did not significantly (*p* > 0.05) change neither in NCD-fed rats nor in HFD STZ rats (Figure 7). 

**Figure 8:**
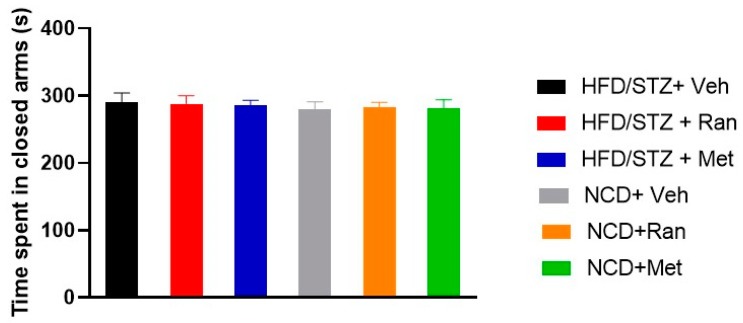
Elevated plus maze (EPM). Bars indicate the time spent in closed arms (s). Data values are means ± SEM. NCD = Normocaloric diet; STZ = Streptozotocin; Veh = Vehicle; HFD = High fat diet; Ran = Ranolazine; Met = Metformin.

Anxiety-like behavior in each experimental group was evaluated by EPM and was not affected by the experimental model or treatment (F_(2,42)_=0.022; *p* = 0.97; *n* = 8 per group; Figure 8). Mean velocity and total distance moved did not differ among groups neither in the FST nor in the EPM (data not shown).

## 4. Discussion

Recent data have associated T2DM with an increased risk of developing cognitive impairment and AD [3,4,7,8,33]. The reasons underlying this increased risk have not been fully elucidated to date, therefore the prevention of cognitive comorbidities in patients with T2DM represents a great therapeutic and research challenge. Ranolazine is an anti-ischemic and anti-anginal drug that increases the time before the occurrence of angina symptoms. Several studies showed that ranolazine has hypoglycemic effects in patients with T2DM [11,12,34,35]. However, the mechanisms underlying these effects have not been completely explained.

In the present study, we investigated the glycometabolic effects of ranolazine in male Wistar rats with type 2 diabetes. HFD/STZ rats developed a disease with several characteristics typical of human T2DM [18]. 

Data from this study demonstrate that 8-weeks treatment with ranolazine is associated with a significant improvement of glycemia in diabetic rats. Animals from the HFD/STZ + Ranolazine group had significantly reduced blood glucose levels during IPGTT after 8 weeks of treatment, compared to baseline (after induction of diabetes) and to diabetic animals from HFD/STZ + Vehicle group. These data are in agreement with a study conducted on mice, demonstrating that ranolazine improved glycemic profiles in diabetic mice, preserved islet morphology, and increased β-cell survival [14]. Interestingly, we also observed reduced IL-6 levels in the HFD/STZ + Ranolazine group, as compared to the HFD/STZ + Vehicle group, suggesting that ranolazine may also ameliorate the T2DM-associated inflammatory profile. 

In this study, we compared the effect of ranolazine with metformin, used as a positive control of an effective anti-hyperglycemic treatment. Data showed that the hypoglycemic effect of ranolazine is almost comparable to that of metformin. 

Furthermore, rats from the HFD/STZ + Ranolazine group showed a statistically significant increase in lean body mass with no significant changes in fat mass. Our data appear to substantiate the results of an in vitro study from Terruzzi et al., in which they demonstrated that ranolazine stimulates myogenesis by activating a calcium signaling pathway [36], even if, at present, we do not have any data on the molecular mechanisms implicated in the lean body mass increase in our experimental setting. Importantly, since in humans, skeletal glucose consumption accounts for 70%–80% of whole-body glucose homeostasis [37], ranolazine ability to increase lean body mass could potentially have an even greater hypoglycemic effect than observed in murine models. 

Our study demonstrates, also, that long term treatment with ranolazine or metformin has protective effects against the development of cognitive decline by preventing the damage of memory (measured by latency time of entry into the dark compartment during a PA test) and working memory (measured by the time spent for the exploration of the familiar and novel object in the nORT). Our results showed that rats treated with ranolazine or metformin are protected from cognitive decline in comparison to diabetic rats from the HFD/STZ + Vehicle group (*p* < 0.001). The data we obtained are consistent with earlier studies that reported the protective effect of metformin against learning and memory deficit [38]. 

Metformin has been suggested to exert positive effects on cognitive decline by acting both indirectly, decreasing blood glucose levels, and directly at the level of CNS [39]. 

Based on the findings of the present study, it may be suggested that ranolazine also has protective effects against memory deficit in diabetic rats, but our data do not allow us to draw any firm conclusion on possible mechanisms of action, even if we obtained evidence of an improved inflammatory profile in ranolazine-treated diabetic animals. Therefore, data from histological studies will be necessary to investigate the possible mechanism underlying ranolazine’s effects.

Finally, we investigated ranolazine’s effects on depressive- and anxious-like behaviors. According to a previous study, we confirmed that the diabetic HFD/STZ model presents a depressive-like behavior [40]. The time of immobility observed in FST in diabetic rats from the HFD/STZ + Vehicle group was significantly increased (*p* < 0.009) in comparison to non-diabetic rats from the control NCD + Vehicle group. The current study demonstrated that treatment with ranolazine reduced the duration of immobility in diabetic rats, attenuating the depressive-like behavior. Metformin had similar effects. No differences were detected among experimental groups in the EPM test for anxiety. 

In our study, we demonstrated that ranolazine has consistent effects against cognitive impairment and depressive-like behavior, which has never been described before. Further studies are required to clarify the molecular mechanisms involved and to evaluate if ranolazine effects are maintained in both sexes, as only male rats were analyzed in the present study. 

In conclusion, our results confirmed the hypoglycemic effect of ranolazine and showed that this drug may exert beneficial actions against cognitive decline and depression. These results could open interesting perspectives for clinical practice.
